# Willingness to participate in genome testing: a survey of public attitudes from Qatar

**DOI:** 10.1038/s10038-020-0806-y

**Published:** 2020-07-28

**Authors:** Hanan F. Abdul Rahim, Said I. Ismail, Amel Hassan, Tasnim Fadl, Salma M. Khaled, Bethany Shockley, Catherine Nasrallah, Yara Qutteina, Engi Elmaghraby, Heba Yasin, Dima Darwish, Khalid A. Fakhro, Radja Badji, Wadha Al-Muftah, Nahla Afifi, Asmaa Althani

**Affiliations:** 1 College of Health Sciences, QU Health, Qatar University, Doha, Qatar; 2 Qatar Genome Program, Qatar Foundation, Qatar Science and Technology Park, Innovation Center, Doha, Qatar; 3 Department of Pediatrics, Sidra Medicine, Doha, Qatar; 4 Social and Economic Survey Research Institute, Qatar University, Doha, Qatar; 5 Department of Human Genetics, Sidra Medicine, Doha, Qatar; 6 Department of Genetic Medicine, Weill Cornell Medical College, Doha, Qatar; 7 College of Health and Life Sciences, Hamad Bin Khalifa University, Doha, Qatar; 8 Qatar Biobank, Qatar Foundation, Doha, Qatar

**Keywords:** Public health, Genetics research, Patient education

## Abstract

Genomics has the potential to revolutionize medical approaches to disease prevention, diagnosis, and treatment, but it does not come without challenges. The success of a national population-based genome program, like the Qatar Genome Program (QGP), depends on the willingness of citizens to donate samples and take up genomic testing services. This study explores public attitudes of the Qatari population toward genetic testing and toward participating in the QGP. A representative sample of 837 adult Qataris was surveyed in May 2016. Approximately 71% of respondents surveyed reported that they were willing to participate in the activities of the QGP. Willingness to participate was significantly associated with basic literacy in genetics, a family history of genetic diseases, and previous experience with genetic testing through premarital screening. Respondents cited the desire to know more about their health status as the principle motivation for participating, while lack of time and information were reported as the most important barriers. With QGP plans to ramp up the scale of its national operation toward more integration into clinical care settings, it is critical to understand public attitudes and their determinants. The results demonstrate public support but also identify the need for more education and individual counseling that not only provide information on the process, challenges, and benefits of genomic testing, but that also address concerns about information security.

## Introduction

Precision health is a new paradigm that is increasing the use of genomic technologies for the assessment of susceptibility to major diseases as well as individual responses to therapeutic regimes [1, 2], thus, increasing the effectiveness of medical intervention. Increasingly, evidence is pointing to the influence of precision health on improved treatment and health outcomes for patients with breast [3], lung [4], and colorectal [5] cancers. Realizing this potential, and aided by the rapid evolution of sequencing technology, several countries have embarked on national projects to characterize the genomes of their own populations in preparation for large-scale implementation in clinical settings [1]. The State of Qatar in the Arabian Gulf is one of those countries. In late 2015, Qatar launched the pilot phase of the Qatar Genome Program (QGP), which is a population-based genome program aiming to sequence the whole genomes for a significant proportion of the Qatari population. The program has a comprehensive plan to facilitate the implementation of precision medicine involving drafting genomic research regulations and policies, building genomic research networks, building local human capacity, and expediting the integration of genomics into the healthcare system. With a native population of around 300,000 citizens, state of the art genomic infrastructure, and a centralized and dynamic health care system, Qatar has all the essential ingredients for becoming a global model in implementing precision medicine.

As a population-based program, QGP relies on community support and engagement, which in turn depends on the public’s trust and willingness to participate. With the end of the pilot phase and in preparation for the subsequent large-scale phases of the program, it was important for QGP to gauge preparedness and potential challenges at the public level. To that end, QGP commissioned the Social and Economic Survey Research Institute (SESRI) in Qatar to conduct the survey. The survey was conducted among a representative sample of the Qatari population to measure public awareness and attitudes related to genetic and genomic testing as well as willingness to participate in QGP activities. The findings of this survey address the dearth of empirical studies on public attitudes toward genetic research and genome-based health care from the Middle East and North Africa (MENA) region, where social, legal, and ethical issues related to genetic testing have been debated. Outcomes of these surveys are needed for research-based guidelines and recommendations to inform QGP’s strategic planning and pave the way for implementation of precision medicine at a national level.

## Population and methods

### Sample

The target population for the survey was Qatari adults, aged 18 years or older. At ~98% coverage [6], Qatar has one of the highest cell phone penetration rates globally [7], making cell phone based sampling an effective and efficient choice for drawing a representative sample of the target population. In this survey, the SESRI of Qatar University, working with local cellular phone providers, drew a representative cell phone sample using a list-based dialing technique [8]. The target sample size for this survey was estimated at 800, calculated using the standard formula for estimating proportions, with significance level set at 5%:

$$n = z^2\frac{{p\left( {1 - p} \right)}}{{e^2}}deff.$$

Following convention, *p* was set at 50.0% to identify the largest sample size requirement [9]. The design effect (*deff*), which reflects the relative efficiency of a statistical estimate based on a complex sample design compared with a sample of the same size selected by simple random sampling, was estimated at 1.1 based on previous phone surveys with the same sample design. Sampling error (*e*) was set at 3.5%, which is a reasonable level of sampling error compared with previous studies in Qatar [10].

### Questionnaire development

Researchers designed a semi-structured questionnaire to collect information related to public perceptions of genetic testing in general and genomic testing in specific. The questionnaire was based on an extensive review of public attitude surveys in a number of countries [11–15], with adaptations to the local context. The questionnaire included closed-ended questions covering demographic characteristics, awareness of genetic and genomic testing, sources of information on genetic and genomic testing, basic literacy in genetics, past experience with genetic testing (for example through premarital testing), family history of chronic diseases, family history of genetic diseases, willingness to participate in QGP genomic activities, and perceived facilitators and barriers to such participation.

Awareness of genetic testing was asked first (*have you ever heard of genetic tests*), and subsequent questions about sources of information for hearing about genetic tests were only asked to those who answered affirmatively. To ask about knowledge of genomic testing, the interviewers used the following script: “*genetic tests that scan an entire person’s genetic makeup for health risks are currently available. Have you heard anything about those tests?*” Basic literacy in genetics was assessed using eight questions (Appendix 1 survey questionnaire). Each question was judged as “correct” or “incorrect” by the taking the standard scientific understanding as a baseline. Correct answers received a score of “1” and incorrect answers a score of “0.” The final score for scientific literacy was calculated by summing the number of correct answers. Questions to measure previous experience with genetic testing gave the respondent a list of examples of such tests, including newborn screening, pre-martial genetic screening, and prenatal testing. The question about willingness to participate in QGP activities was asked after giving respondents a description of QGP aims. Additional open-ended questions about facilitators and barriers to participation in QGP activities allowed respondents to express in their own words responses that were not anticipated by the researchers.

Questions were initially written in English. A professional translator translated them into Arabic, and bilingual researchers carefully checked the translations. The questionnaire was pilot-tested on 30 respondents (not included in the final study) to ensure clarity and understandability of all terms and questions to a layperson. Pretest results were used to refine question wording, response categories, introduction script, transitions, interviewer instructions, and interview length.

### Survey administration

The public opinion survey was conducted in May 2016 using the Computer Assisted Telephone Interview module of the survey management software BLAISE (Statistics Netherlands). Twenty-three experienced Arabic-speaking survey interviewers, who are long-term residents of Qatar, with university or at least above secondary education were trained both on the protocol for conducting phone interviews and on the survey questions. To maximize the likelihood of reaching respondents, interviewers made calls over different times of the day across different days of the week.

Cellular phone numbers of respondents were released to interviewers in batches to ensure that call procedures were followed per protocol for all numbers. The use of batches also improved the representativeness of the survey by balancing the distribution of phone numbers across respondent characteristics. For every phone number in the sample, there were at least six attempts to complete the interview. Phone numbers with break-offs and soft refusals were transferred to dedicated interviewers with advanced experience.

Interviewers obtained informed consent from potential responders by first introducing themselves and the subject of the survey and then explaining the voluntary nature of participation and the confidentiality of responses. Interviewees were given the opportunity to ask any clarification questions before agreeing to take part in the survey.

### Statistical analysis

Data from the surveys were analyzed using STATA version 14.0. Survey weights were used in the calculation of reported percentages, bivariate and multivariate analyses performed. Weights were constructed from the sample selection probability (base weights), adjustment factor to account for nonresponse, and post-stratification calibration. Probabilities (*p* values) for statistical significance (5.0%) were based on the design-based *F* tests, which is a corrected weighted Pearson chi square statistic.

## Results

### Response rate

The response rate in the public opinion survey was 53% (837 respondents out of an estimated eligible 1551 individuals). To reach that number of eligible interviews, 5250 calls were made in total, out of which 3699 were not eligible (not Qatari individuals 18+ years).

### Respondents’ demographics

The demographic characteristics of respondents are shown in Table 1. Approximately one-half of the respondents (49.2%) were male, and the average age of respondents was ~36 years (range 18–75). Over one-half (58.1%) of respondents were married, 45.1% of those marriages were consanguineous, and 89% of married respondents reported having children. Almost 80% of the sample reported a secondary educational level or higher, and approximately one-third of respondents (34.0%) reported a monthly household income of 70 000 QAR (19230 USD) or higher.

**Table 1:** Demographic characteristics of public opinion survey respondents

	%^a^	CI%	N
Male	49.2	45.6–52.7	478
Age			
Mean years (sd)	35.7 (12.7)	34.6–36.7	804
Married	58.1	54.6–61.6	531
Blood related to spouse	45.1	40.8–49.5	241
Has children	89	86.2–91.2	523
Education (highest level)			
Less than secondary	20.4	17.6–23.5	159
Secondary or vocational	47.5	44.0–51.0	386
Undergraduate degree or above	32.1	29.0–35.4	289
Monthly household income^b^			
Less than ~$8,240	23.1	20.0–26.5	159
~$8,240–$13,730	22.9	19.9–26.1	180
~$13,730–$19,230	20.1	17.3–23.2	152
More than ~$19,230	34	30.5–37.6	251
Total sample size^c^	—	—	834

^a^Reported percentages were calculated using survey weights and therefore differ from the raw percentages. The number of respondents reported for each variable corresponds to the unweighted sample

^b^Categories reported have been converted to USD. Income categories were reported in Qatari Riyals; 92 observations are missing in this variable

^†^Three cases missing from this table due to missing values on key variables

### Respondents’ knowledge, attitudes, and willingness

Information about genetic or genomic tests, basic literacy in genetics, past exposure, and family history of disease, including genetic disease, are presented in Table 2. These factors are hypothesized to influence interest in genetic or genomic testing, which may in turn affect willingness to undergo genomic tests. Almost one-half of respondents (51.2%) had heard of genetic testing, while only 27.8% reported having heard of genomic tests. Respondents who completed higher levels of education, including university degrees and above, were more likely to have heard of genetic tests compared with those with lower level of education (less than high school; *p* = 0.0169; data not shown). For genetic testing, social media (22.3%) and word of mouth (22.6%) were the most commonly reported individual sources of information, while only 9.0% of respondents reported that physicians were their main source of information on genetic testing. Books, magazines, and brochures were grouped into one category, accounting for 31.4% of responses. For genomic testing, 13.0% of respondents reported that physicians were the main source of information, compared with 30.3% for word of mouth and 20.3% for social media. More than half of the respondents (56.1%) were able to answer at least 5 out of 8 genetics-related questions correctly and were classified as having a “high” level of basic literacy in genetics.

**Table 2:** Information on and experience with genetic testing and willingness to get tested

	%^a^	CI%	*N*
Heard about genomic testing	27.8	24.8–31.0	245
Source of information on genomic testing^b^			
Doctor	13.0	10.5–19.4	35
Word of mouth	30.3	23.1–34.4	69
Website or social media	20.3	16.7–27.0	52
Traditonal media (TV/radio)	10.4	6.7–14.3	24
Newspapers/magazines/brochures/other	26.0	20.8–31.8	63
Heard about genetic testing	51.2	48.2–55.3	447
Source of information on genetic testing^c^			
Doctor	9.0	7.5–13.0	44
Word of mouth	22.6	18.7–26.4	99
Website or social media	22.3	19.7–27.6	104
Traditonal media (TV/radio)	14.7	11.1–17.5	62
Newspapers/magazines/brochures/other	31.4	26.3–34.8	135
Basic literacy in genetics^d^			
Low	43.9	41.2–48.0	373
High	56.1	52.0–58.8	464
Past experience of genetic testing by a family member			
Carrier testing	39.3	34.7–44.2	172
Prenatal/newborn testing	3.4	2.0–5.8	14
Diagnostic testing	1.4	0.6–3.0	7
None	51.2	46.4–56.0	239
Family history of chronic disease			
Hypertension	47.1	43.5–50.6	382
Obesity	19.6	16.9–22.6	156
Diabetes	62.1	58.6–65.5	520
Cardiovascular disease	19.5	16.8–22.5	160
Stroke	3.9	2.7–5.4	130
Cancer	12.4	10.1–15.0	97
None	22.9	20.1–26.0	191
Family history of genetic disorders	20.4	17.7–23.4	175
Willingness to paritcipate in QGP genomic testing	70.9	67.5–74.1	584

^a^Reported percentages were calculated using survey weights and therefore differ from the raw percentages. The number of respondents reported for each variable corresponds to the unweighted sample

^b^Two cases are missing observations for sources of information on genomic testing; three cases are missing observations for sources of information on genetic testing

^c^Basic literacy in genetics was decided based on the number of correct responses to a set of 8 questions; Literacy of respondents who answered 5 or more questions correctly was labeled “high” and literacy of respondents with less than 5 correct answers was labeled “low”

Respondents reported that their most common experience with genetic testing by a family member was carrier testing (premarital testing). Two-thirds of the respondents (62.1%) reported a family history of diabetes, 12.4% a family history of cancer, and 20.4% reported a family history of genetic disorders.

In all, when given a description of the QGP and its aims, 70.9% of respondents indicated their willingness to participate.

Demographic factors, including age, marital status, consanguinity, and education were not significantly associated with willingness to participate in genomic testing (Supplementary Table 1). Only one category of monthly income (8240 Qatari Riyals −21,3730 USD) was significantly associated with willingness to participate in genomic testing (82%). Table 3 presents the willingness of respondents to participate in genomic testing by a number of non-demographic factors, which were hypothesized to determine attitudes. Of those factors, basic literacy in genetics, previous experience with genetic testing, and a family history of genetic disorders were significantly and positively associated with higher willingness to participate in genomic testing through QGP. Among respondents with a high level of basic literacy in genetics, 76.4% expressed willingness to participate in QGP activities compared with 63.9% of respondents with low levels of basic literacy in genetics. Almost two-thirds (67.3%) of respondents who reported no experience of genetic testing by a family member were willing to participate in genomic testing compared with 76.1% of those who did. Similarly, almost two-thirds (68.6%) of respondents reporting no family history of genetic disease said they were willing to participate in genomic testing compared with almost 80% of respondents with a family history of genetic disease. A history of chronic disease in the family was positively – though not significantly—associated with willingness. Among respondents reporting no family history of chronic disease, 64.9% were willing to participate in genomic testing compared with 72.6% of respondents with a family history of chronic disease. Having previously heard of genetic or genomic tests was not significantly associated with willingness to participate, and the source of hearing about those tests was also not significantly associated with willingness (data not shown).

**Table 3:** Public willingness to participate in the genome project by key experiences and attitudes

	% Willing to participate in QGP^a^	n/N^b^	Design-based F (DF)^c^	*P* value
Heard about genomic testing				
Yes	73.1	178/241	0.719 (1,812)	0.397
No	70	406/575		
Heard about genetic testing				
Yes	73.4	323/435	2.435 (1,812)	0.119
No	68.2	261/381		
Basic literacy in genetics				
Low	63.9	239/362	9.837 (1,812)	0.002
High	76.4	345/454		
Past experience of genetic testing by a family member				
None	67.3	319/470	6.777 (1,812)	0.0094
Some	76.1	265/346		
Family history of chronic disease				
None	64.9	123/185	3.691 (1,812)	0.0551
Some	72.6	461/631		
Family history of genetic disorders				
None	68.6	449/645	7.737 (1,812)	0.0055
Some	79.8	135/171		

^a^Reported percentages were calculated using survey weights and therefore differ from the raw percentages

^b^The number of respondents reported for each variable corresponds to the unweighted sample. N refers to the total number of respondents in the column while n refers to number of observations in the specific cell

^c^Design-based F is a corrected weighted Pearson chi square statistic

### Reasons for willingness or not to participate in genomic testing

**Fig. 1:**
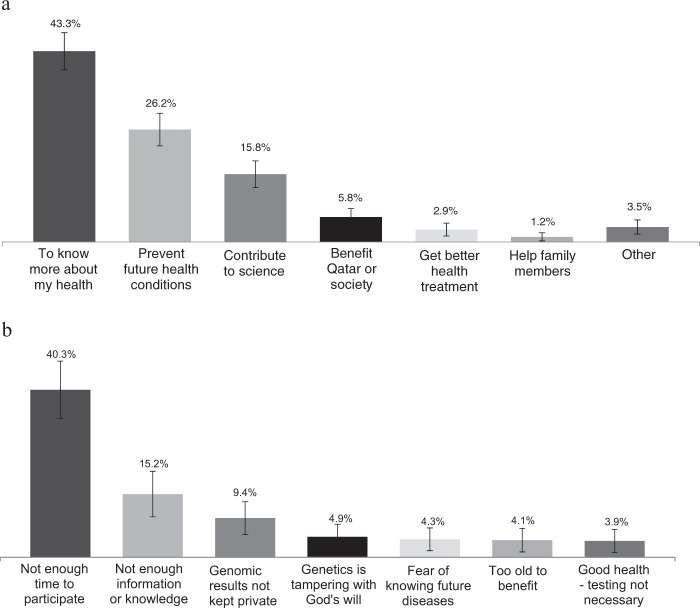
**a** Most important reason for willingness to participate in QGP genomic testing. *Based on the question asked only to respondents who indicated they would be willing to participate in QGP genomic testing (*n* = 584). **b** Most common reasons for unwillingness to participate in QGP genomic testing. *Based on the question asked only to respondents who indicated they would not be willing to participate in QGP genomic testing. Respondents were allowed to give more than one answer (*n* = 232)

Respondents who indicated their willingness to participate in QGP genomic testing were asked for the most important reason for their choice. The top three reasons for willingness to participate were: “*to know more about my health”* (43.3%), “*to prevent future health conditions*” (26.2%), and “*to contribute to science”* (15.8%) (Fig. 1a).

Respondents who indicated that they would not be willing to participate in genomic testing through QGP were asked for the most important reasons for their refusal (Fig. 1b). The most frequently cited explanations for not willing to participate, included “*not enough time to participate*” (40.3%), “*not having enough information or knowledge*” (15.2%), and “*concerns that genomic results not kept private”* (9.4%). Less common reasons were “*concerns that genetics is tampering with God’s will*” (4.9%), and “*fear of knowing future diseases”* (4.3%). The latter respondents often mentioned that the future was “best left up to God to decide.”

## Discussion

To our knowledge, this study is the first population-based survey of public attitudes toward genetic testing and genomic testing in the MENA region. Initially, respondents were asked about their awareness of genetic testing, and we observed that slightly more than half of the respondents had previously heard about genetic tests. We found that attitudes toward genetic testing were generally positive, with the majority expressing their willingness to participate in the initiatives of QGP that include, but are not limited to, promoting and encouraging genomic research. The positive attitudes toward genetic testing are consistent with survey findings reported from the Netherlands [11, 12], United States [13, 14], and Canada [15], where genetic testing is more common. The positive attitudes and willingness to participate are of particular interest, given the concerns that a highly endogamous population would be opposed to genetic testing on cultural grounds and fear of stigmatization if associated with a genetic disease [16, 17]. Nevertheless, there is need for in-depth qualitative research looking at the public’s beliefs and perspectives on genomic testing that may yield questions and concerns not easily identified in a survey.

In our survey, willingness to participate in QGP activities was high despite a relatively low level of awareness of genomic testing. Respondents who indicated their willingness to participate in genomic testing gave as the most important reason their desire to know more about their health. This finding indicates that QGP should have a clear strategy and policy with regard to returning results and educating the public and what those results mean in the short- and long-term for their health and wellbeing. In addition to personal motivations, a combined 21.6% of respondents said they would be willing to participate for altruistic reasons, namely contributing to scientific research and to improve Qatar or Qatari society, indicating that nationalistic attitudes play a secondary role to concerns about personal health benefits in motivating participation (Fig. 1a). Understanding the drivers and motivations is important for shaping awareness raising campaigns and educational messages.

In this survey, respondents were asked to report on any reason that would choose not to participate in QGP genomic testing. Almost half (55.5%) of responses were barriers of a practical rather than attitudinal nature, including not having enough time or having inadequate information about the program. At the same time, almost 10% of responses were related to concerns about the confidentiality of results. This concern has been reported in a number of studies [13, 14] in populations where genetic testing is more widespread. While QGP should conduct general informational campaigns about the procedure and time involved in genomic testing, it also needs to communicate clearly to the public the systems and measures in place to protect patient information. Such communications need to be explicit, user-friendly, and appropriate to different levels of education.

We had originally anticipated that willingness to participate in genomic testing might be influenced by familiarity and past experiences with genetic testing. Among respondents, the most common experience with genetic testing was carrier testing, which is expected given that the law in Qatar requires nationals to take part in premarital genetic testing. This finding is of interest as it suggests that premarital genetic testing may be a good platform to introduce information about genomic testing to the public. This approach suggests the need for involving healthcare professionals and health educators in educating the public on the benefits of genomic analysis. Just as the success of premarital testing depended greatly on religious support for its success, genomic testing also needs to engage with key stakeholders, including healthcare providers and religious scholars [18, 19].

The survey has several strengths, including a representative population-based sample of Qatari nationals and robust survey methods, which emphasized careful interviewer training to standardize questionnaire administration and to ensure clarity and salience of terminology in the respondents’ native language (Arabic). That said, it is important to recognize that social desirability is difficult to avoid in attitudinal surveys. Respondents sometimes say “yes” to questions posed by interviewers or agree with statements when their true feelings are less positive. This social desirability bias can arise in spite of the best efforts of survey designers and interviewers to assure the respondents that their true opinions are valuable. Thus, the true level of willingness may be inflated by respondent desires to offer what they believe to be the socially acceptable answer to the question. Another limitation of this study is the response rate, which is a challenge in phone surveys generally. Interviewers were trained to call make repeated attempts to reach the sample population, and cases that were difficult to recruit were referred to the most experienced interviewers.

Relatedly, even if these findings do reflect the true nature of attitudes about the QGP, it is more difficult to get respondents to engage in a certain behavior (such as giving a blood sample) than to answer affirmatively to a survey questions. The strong levels of support found in this survey should be interpreted as an indication that the majority of respondents feel positively inclined toward participation. Subsequently, QGP should capitalize on that good will by employing engagement programs to raise public knowledge thereby widening participation. All in all, it is clear that there is substantial positive attitudes toward the QGP, yet more efforts need to be directed toward educational initiatives on genomics for the public.

In conclusion, the absence of deep-rooted cultural or ideological objections to genetic testing and genomic testing are very encouraging for nascent programs exploring genomic testing in understudied global populations. However, this enthusiasm needs to be balanced by a sincere effort to raise public awareness to quell shared fears and ensure that public discourse and introduction of genomics program is done in a culturally sensitive and appropriate manner.

## Supplementary information

Appendix 1 (Questionnaire)

Supplementary Table
