# Supplementary material for: Willingness to participate in genome testing: a survey of public attitudes from Qatar
Source: J Hum Genet. 2020 Jul 28;65(12):1067–73. doi: 10.1038/s10038-020-0806-y (PMC7605429; doi:10.1038/s10038-020-0806-y)
Supplement: Supplementary file 1 — Appendix 1 (Questionnaire) [file 10038_2020_806_MOESM1_ESM.docx]

**Qatar Genome Project Study**

**Survey of Public Attitudes**

**Description**

Qatar Genome Project (QGP) has commissioned the Social and Economic Survey Research Institute (SESRI) at Qatar University to conduct a public survey of 800 Qatari nationals exploring knowledge and attitudes towards genetic research (specifically genome mapping) and support for the QGP.

{Q: INTRO}

Good morning/afternoon/evening, the Qatar Genome Project in cooperation with Qatar University’s Social and Economic Survey Research Institute is conducting an important survey to understand public views about health research in Qatar. You have been randomly chosen to participate in this survey. Your assistance will help us to better understand this important topic.

My name is ___ and if you have no questions we can get started!

INTERVIEWER, IF NECESSARY: We can assure you that all of your answers will be kept confidential and will be released only as statistics in which no individual can be identified

1 QUESTIONNAIRE 5 REFUSAL

2 APPOINTMENT 6 ANSWERING SERVICE

3 NO ANSWER 7 WRONG NUMBER/DISCONNECT

4 BUSY 8 INELIGIBLE / OTHERS

**PROGRAMMER: PLEASE RECORD LANGUAGE OF INTERVIEW FROM BLAISE**

{Q: SAFE}

For your safety, if you are currently doing anything that would require your full attention, such as driving or operating heavy equipment, then I’ll need to call you back at a time convenient for you.

IV: IF R SEEMS TO BE IN SITUATION WHERE CANNOT HEAR CLEARLY OR SPEAK WITHOUT INFLUENCE FROM OTHERS, ASK:

Are you able to answer questions without distraction and in conditions that are comfortable to you?

- - 1. GO ON
    2. R CALLBACK

1. DON’T KNOW
2. REFUSED

{Q: CONFIRM}

Second, I need to confirm that you are at least 18 years of age and live in the State of Qatar.

[**IF NECESSARY SAY**: Your answers are confidential, and we don’t use anybody’s name.]

1. R IS RESIDENT ADULT, PROCEED
2. R IS NOT ADULT (SKIP THANKS 2)
3. R IS NOT QATAR RESIDENT (SKIP THANKS 2)

{Q: CONFDNTL}

Before we begin let me reassure you that results from the survey will be presented as statistical summaries in which no individual can be identified, and you may choose not to answer any question at any time. If you have no further questions, let’s get started!

IF NECESSARY: We're calling from Qatar University conducting a survey that will explore the public views about genetic research in Qatar.

IF ASKED: The survey will take about 20 minutes and you can complete it in several parts if you need to leave at any time. All of your answers will be saved and we’ll resume right where you left off at a time that is convenient for you.

IF NECESSARY: SOCIAL UTILITY MESSAGE:

The Social and Economic Survey Research Institute is a research unit of Qatar University providing scientifically valid survey research in Qatar and the region. We are subject to oversight by an institutional review board and comply with the standards of the survey research profession worldwide. We do not report any individual answers, and strictly protect your confidentiality. In addition, we randomly select respondents so that everyone has an equal chance of selection. Consequently, you represent at least 500 of people in Qatar, so your participation is very important to provide the perspective of other people just like you. By answering a few questions you can make this study more representative and therefore have more impact.

1. GO ON
2. R CALLBACK
3. **DEMOGRAPHICS**

PROGRAMMER: Retrieve RespSample from the sample. Initialize Resptype=0.

{Q: CITIZEN}

Are you Qatari or a non-Qatari?

1. YES, QATARI CITIZEN
2. NO, NOT QATARI CITIZEN
3. HAS TEMPORARY QATARI “MISSION” PASSPORT
4. WATHIQA/OTHER (SPECIFY)
5. DON’T KNOW
6. REFUSED

PROGRAMMER:

***IF CITIZEN = DON’T KNOW/REFUSED SKIP TO ELIGREF***

***IF (CITIZEN = 1 OR CITIZEN =4) AND RESPSAMPLE =1, RESPTYPE = 1***

{Q: CHECKCIT}

**IF CITIZEN = 1 OR CITIZEN =4 AND RESPSAMPLE <> 1 ASK**

[INTERVIWER: FOR CLARIFICATION PURPOSE, ASK IF THE RESPONDENT HAS A QATARI PASSPORT]

1        HAVE QATARI PASSPORT

2        DON’T HAVE QATARI PASSPORT

3        HAS TEMPORARY QATARI “MISSION” PASSPORT

8        DON’T KNOW

9        REFUSED

***PROGRAMMER:***

***IF CHECKCIT= DON’T KNOW/REFUSED SKIP TO ELIGREF***

***IF CHECKCIT = 1, RESPTYPE = 1***

***IF RESPTYPE <> 1, EXIT TO THANKS2***

{Q: ELIGREF}

PROGRAMMER: IF CITIZEN OR CHECKCIT = DK OR REF, ASK:

Thank you for your time. Unfortunately, we need the answer to this question in order to proceed with the survey. We hope you will reconsider given the importance of the information to residents and visitors. All answers to this and all questions are presented as grouped data and no individual’s information is revealed. Have a nice day. If you need more reassurance please check our website at sesri.qu.edu.qa or email us at [surveys@qu.edu.qa](mailto:surveys@qu.edu.qa) . We can also send you an SMS text if you would like.

INTERVIEWER: IF CHANGES MIND, GO BACK AND GET ANSWER PROGRAMMER: SKIP TO REFUSAL DISPOSITIONS

{Q: GENDER}

**If RESPTYPE = 1, ASK**

[INTERVIEWER: ENTER REPONDENT’S GENDER]

IF UNCERTAIN, SAY: The survey requires you to tell me your gender.

1. MALE
2. FEMALE

{Q: MARITAL}

What is your current marital status? Are you married, separated, divorced, widowed, or have you never been married?

1 Married

2 Separated

3 Divorced

4 Widowed

5 Never married

8 DON’T KNOW

9 REFUSED

***If MARITAL=1 AND GENDER=3, ASK***

{Q: WOMAN}

Are you currently married to more than one woman?

1. YES
2. NO
3. DON’T KNOW
4. REFUSED

***IF WOMAN=1, ASK***

{Q: WOMAN1}

Are any of them related to you by blood?

1. YES
2. NO
3. DON’T KNOW
4. REFUSED

***If WOMAN1=1, ASK***

{Q: NUMBER}

How many are related to you by blood?

1. 1
2. 2
3. 3
4. 4
5. DON’T KNOW
6. REFUSED

***If WOMAN1=1, ASK***

{Q: RELATION1}

What is the type of relationship? [SELECT ALL THAT APPLY]

1. FIRST COUSIN ON FATHER’S SIDE
2. FIRST COUSIN ON MOTHER’S SIDE
3. SECOND COUSIN ON FATHER’S SIDE
4. SECOND COUSIN ON MOTHER’S SIDE
5. OTHER BLOOD RELATIVE (SPECIFY)
6. DON’T KNOW
7. REFUSED

***If MARITAL=1 AND GENDER=2 OR WOMAN=2, ASK***

{Q: CONSANG}

Are you related by blood to your spouse?

1. YES
2. NO
3. DON’T KNOW
4. REFUSED

***If CONSANG=1, ASK***

{Q: RELATION}

What is the type of relationship? (Not multiple selection)

1. FIRST COUSIN ON FATHER’S SIDE
2. FIRST COUSIN ON MOTHER’S SIDE
3. SECOND COUSIN ON FATHER’S SIDE
4. SECOND COUSIN ON MOTHER’S SIDE
5. OTHER BLOOD RELATIVE (SPECIFY)
6. DON’T KNOW
7. REFUSED

{Q: CHILDREN}

***If MARITAL<5, ASK***

Do you have children?

1. YES
2. NO
3. DON’T KNOW
4. REFUSED

{Q: EDUC1}

What is the highest level of education you have completed?

[INTERVIEWER: PROBE FROM CATEGORIES / READ IF NECESSARY]

- - - 1. NEVER ATTENDED ANY SCHOOL
      2. PRIMARY (1-6)
      3. PREPARATORY (7-9)
      4. Vocational
      5. Secondary (10-12)
      6. Post secondary (ex: Diploma)
      7. University graduate/BA/BCom/BSc
      8. Master’s degree
      9. Ph.D/m.d
      10. OTHER (SPECIFY)

1. DON’T KNOW
2. REFUSED

{Q: EMPLOY}

Are you currently working?

1. YES
2. NO
3. DON’T KNOW
4. REFUSED

{Q: AGE1}

In which year you were born?

____________________________

1. DON'T KNOW
2. REFUSED

{Q: AGE2}

**If AGE=9998 OR 9999, ASK**

What is your age range?

1. 18 – 24 years
2. 25 - 34 years
3. 35 - 44 years
4. 45 - 54 years
5. 55 - 64 years
6. 65 years or more
7. DON’T KNOW
8. REFUSED
9. **KNOWLEDGE/LITERACY**

Now I’m going to ask you a few questions related to genetics and genetic testing.

{Q: HEARGEN}

Have you ever heard of genetic tests?

1. YES
2. NO
3. REFUSED

**IF HEARGEN=1 ASK**

{Q: SOURCE1}

What was your main source of information about genetic tests? [CHOOSE ONE ONLY]

1. Newspapers, either online or print
2. Magazine, either online or print
3. Websites or Blogs on internet
4. Social media, such as Facebook, Twitter, Instagram, or Snapchat
5. Books
6. Brochures, pamphlets or other similar materials
7. Television
8. Radio
9. Your physician
10. Word of mouth such as family, friends or colleagues
11. Or something else (SPECIFY)?
12. Don’t Know
13. Refused

{Q: GENOME}

Genetic tests that scan an entire person’s genetic makeup for health risks are currently available. Have you heard anything about these tests?

1. Yes
2. No
3. DON’T KNOW
4. REFUSED

{Q: SOURCE2}

What was your main source of information about these tests? [CHOOSE ONE ONLY]

1. Newspapers, either online or print
2. Magazine, either online or print
3. Websites or Blogs on internet
4. Social media, such as Facebook, Twitter, Instagram, or Snapchat
5. Books
6. Brochures, pamphlets or other similar materials
7. Television
8. Radio
9. Your physician
10. Word of mouth such as family, friends or colleagues
11. Or something else (SPECIFY)?
12. Don’t Know
13. Refused

**LITERACY/ BASIC UNDERSTANDING**

Now I am going to read out to you a number of statements about genetics and genetic testing. I would like you to tell me if you think these statements are true or false.

{Q: LITERACY1}

If two people are from the same ethnicity, they will be more genetically similar to each other than two people from different ethnicities

INTERVIEWER, IF NEEDED, SAY: We mean people from the same ethnic group or common origins.

1. True
2. False
3. DON’T KNOW
4. REFUSED

{Q: LITERACY2}

You have more genes in common with your brother or sister than with your cousins

1. True
2. False
3. DON’T KNOW
4. REFUSED

{Q: LITERACY3}

People share more genes with their paternal cousins than their maternal cousins

1. True
2. False
3. DON’T KNOW
4. REFUSED

{Q: LITERACY4}

The sex of the baby is determined by the father

1. True
2. False
3. DON’T KNOW
4. REFUSED

{Q: LITERACY5}

Every trait is controlled by a specific single gene (example: height is controlled by a height gene, eye color is controlled by an eye color gene)

1. True
2. False
3. DON’T KNOW
4. REFUSED

{Q: LITERACY6}

We can only say that a disease is genetic if it has affected more than one family member

1. True
2. False
3. DON’T KNOW
4. REFUSED

{Q: LITERACY7}

Some genetic diseases appear later in adult life rather than appearing in childhood

1. True
2. False
3. DON’T KNOW
4. REFUSED

{Q: LITERACY8}

Can human health habits affect the severity of how some genetic diseases emerge?

1. True
2. False
3. DON’T KNOW
4. REFUSED

{Q: FAMILY}

To your knowledge, do any of your immediate family members have any of the following?

We mean fathers, mothers, children, brothers or sisters.

(SELECT ALL THAT APPLY)

1. Diabetes
2. Stroke
3. Hypertension
4. Cancer
5. Cardiovascular diseases
6. Obesity
7. NONE
8. DON’T KNOW
9. REFUSED

{Q: FAMILY1}

To your knowledge, do any of your immediate family members have any of the following? (SELECT ALL THAT APPLY)

1. Down’s syndrome
2. Mental retardation, autism, or developmental delay
3. Blood disorder, such as hemophilia or sickle cell
4. Muscular dystrophy or neuromuscular disease
5. Skeletal disorder, like dwarfism or other bone abnormalities
6. Huntington disease or other adult neurological diseases (e.g., dementia, Alzheimer’s)
7. Heart defect
8. Cleft lip/cleft palate
9. Any birth defect not in this list
10. Any other genetic diseases that you recall? (SPECIFY)
11. NONE
12. DON’T KNOW
13. REFUSED
14. **BEHAVIORS**

**Now we’re going to ask about your experience with genetic testing**

{Q: GENTEST}

**[PROGRAMMER: CHOICE 4 ONLY FOR FEMALES: IF GENDER=5**

Have you ever done any of the following genetic testing? (SELECT ALL THAT APPLY)

1. Newborn screening
2. Diagnostic testing
3. Carrier testing including pre-marital genetic testing
4. Prenatal testing
5. Preimplantation testing for women or In Vitro Fertilization (IVF)
6. Predictive and pre-symptomatic testing
7. Forensic testing
8. NONE OF THESE
9. DON’T KNOW
10. REFUSED

**IF GENTEST=8, ASK**

{Q: GENTEST1}

Have any of your immediate family members ever done any of the following genetic testing? (SELECT ALL THAT APPLY)

1. Newborn screening
2. Diagnostic testing
3. Carrier testing including pre-marital genetic testing
4. Prenatal testing
5. Preimplantation testing for women or In Vitro Fertilization (IVF)
6. Predictive and pre-symptomatic testing
7. Forensic testing
8. NONE OF THESE
9. DON’T KNOW
10. REFUSED

{Q: STUDY}

Have you ever been asked to participate in a research study about health?

1. YES
2. NO
3. DON’T KNOW
4. REFUSED

**IF STUDY=1, ASK**

{Q: PARTICIPATE}

Did you participate?

1. YES
2. NO
3. REFUSED

**IF PARTICIPATE =2, ASK**

{Q: NREASON}

What was the main reason for not taking part in the health study?

1. Not interested
2. Worried about how the information might be revealed or used by others
3. Worried about how the information might affect me
4. Worried about what the information might mean for my relatives
5. Worried about what the information might mean for my society
6. It will not have an effect on my life in any way
7. OTHER (SPECIFY)
8. DON’T KNOW
9. REFUSED

**IF PARTICIPATE =1, ASK**

{Q: TYPE}

Please specify the type of participation (SELECT ALL THAT APPLY)

1. Filling a questionnaire
2. Taking medication
3. Being interviewed
4. Being physically examined
5. Providing a blood a blood sample
6. Providing a tissue sample
7. Other (specify)
8. DON’T KNOW
9. REFUSED
10. **ATTITUDES**

**ATTITUDES/BELIEFS ABOUT GENETIC TESTING IN GENERAL**

[Q: ATTITUDES1-6]

We’d like to ask your overall view of genetic testing. For each of the following statements, please tell me whether you agree or disagree.

[PROGRAMMER: PLACE ORDER OF SELECTION IN SAME ORDER AS READ TO REDUCE ERROR]

|  | Agree | Disagree | NEITHER AGREE NOR DISAGREE |
| --- | --- | --- | --- |
| 1. I believe genetic testing can improve the quality of life and health |  |  |  |
| 2. Genetic testing does more good than harm |  |  |  |
| 3. Genetic testing is tampering with the will of God |  |  |  |
| 4. Genetic testing is useful for informing the marriage decisions based on knowledge of genetic predisposition for disease |  |  |  |
| 5. Fear of the results becoming available to others may make me hesitant to do genetic testing |  |  |  |
| 6.Society considers people who have genetic disorders to be inferior |  |  |  |

**ATTITUDES ABOUT THE PURPOSE OF GENETIC TESTING**

[Q: PURPOSE1-9]

Do you approve or disapprove of using genetic testing for the following purposes?

For each purpose, please tell me whether you strongly approve, approve, disapprove, or strongly disapprove.

[PROGRAMMER: PLACE ORDER OF SELECTION IN SAME ORDER AS READ TO REDUCE ERROR]

| **Purpose** | Strongly Approve | Approve | Disapprove | Strongly Disapprove | UNSURE |
| --- | --- | --- | --- | --- | --- |
| 1. For optimizing medical treatment |  |  |  |  |  |
| 2. For solving crimes |  |  |  |  |  |
| 3. For determining lineage (family birth line, ancestors) |  |  |  |  |  |
| 4. For pre-marital health counseling |  |  |  |  |  |
| 5. For determining likelihood of getting a disease |  |  |  |  |  |
| 6.For diagnosis of rare diseases |  |  |  |  |  |
| 7.For diagnosis of common diseases, such as diabetes |  |  |  |  |  |
| 8. For predicting future health complications for people who already have a disease |  |  |  |  |  |
| 9. For research purposes |  |  |  |  |  |

**USE AND AVAILABILITY**

[Q: USE1-4]

For each of the following statements, please tell me whether you agree or disagree.

[PROGRAMMER: PLACE ORDER OF SELECTION IN SAME ORDER AS READ TO REDUCE ERROR]

|  | Agree | Disagree | NEITHER AGREE NOR DISAGREE |
| --- | --- | --- | --- |
| 1. The use of genetic tests among people should be promoted |  |  |  |
| 2. Genetic tests should be available for those who want to use them |  |  |  |
| 3. Prenatal genetic tests should be offered to all pregnant women |  |  |  |
| 4. People should be preventively tested for all kinds of diseases (both hereditary and non-hereditary) |  |  |  |

**WILLINGNESS & PERSONAL RELEVANCE**

For this section we want to assess how much relevance these test might have for you personally. In other words we will try to understand what *you* want to know and what *you* would do with the results.

[Q: RELEVANCE1-9]

For each of the following statements, please tell me whether you strongly agree, agree, disagree, or strongly disagree.

**[PROGRAMMER: PLACE ORDER OF SELECTION IN SAME ORDER AS READ TO REDUCE ERROR]**

|  | Strongly Agree | Agree | Disagree | Strongly Disagree | NEITHER AGREE NOR DISAGREE |
| --- | --- | --- | --- | --- | --- |
| 1. I would want to know about my genetic make-up |  |  |  |  |  |
| 2. I do NOT want to know what kind of disease I could get in the future |  |  |  |  |  |
| 3. I only want to be genetically tested for predisposition of diseases that can be cured |  |  |  |  |  |
| 4. I only want to be genetically tested for predisposition of a disease if I can prevent this disease |  |  |  |  |  |
| 5. I want a genetic test for my risk of developing a cardiovascular disease |  |  |  |  |  |
| 6. I want a genetic test for my risk of developing cancer |  |  |  |  |  |
| 7. I want a genetic test for my risk of developing diabetes |  |  |  |  |  |
| 8. I want a genetic test for my risk of developing dementia |  |  |  |  |  |
| 9.I would want the results of my genetic test to be kept for my future health needs |  |  |  |  |  |

1. **The Qatar Genome Project (QGP) and perceived benefits to Qatar**

The Qatar Genome Project is a national project initiated by Qatar Foundation. The project aims to use the latest technological developments in the field of genomics and employ them in providing better health care. The project is in its pilot phase and seeks to create a database of genetic information for the Qatari community. The project will help implement the concept of personalized medicine in healthcare in Qatar improving preventive medicine.

**c. Willingness to participate**

[Q: WILLING]

Having read the description of the project, would you be willing to participate in the Qatar Genome Project?

1. YES
2. NO
3. DON’T KNOW
4. REFUSED

[Q: WHYNOT]

**IF WILLING=2, ASK**

What would you say is the reason why you would choose not to participate?

**INTERVIEWER: PROBE FROM RESPONSE AND SELECT UP TO THREE ANSWERS (“Any other reason?”) IN ORDER OF MENTION BY RESPONDENT**

1. HAVING AN EXTRA TUBE OF BLOOD DRAWN
2. KEEPING MY INFORMATION PRIVATE AND SECURE
3. INTERFERING WITH THE WILL OF GOD BY TAKING PART IN THE COLLECTION OF GENETIC DATA
4. REVEALING MY PERSONAL PREDISPOSITION TO DISEASES THAT COULD APPLY TO OTHER FAMILY MEMBER
5. QATAR IS A SMALL PLACE. MY INFORMATION IS BOUND TO GET OUT.
6. CREATING TENSION BETWEEN MY FAMILY MEMBERS
7. DON’T HAVE ENOUGH TIME
8. DON’T HAVE ENOUGH KNOWLEDGE
9. OTHER (PLEASE SPECIFY)
10. DON’T KNOW
11. REFUSED

**IF WILLING=1, ASK**

[Q: WHYYES]

What would you say is the most important reason why you would choose to participate?

**INTERVIEWER: PROBE FROM RESPONSE**

1. TO KNOW MORE ABOUT MYSELF AND MY HEALTH
2. CONTRIBUTION TO SCIENCE
3. TO PREVENT ANY CONDITIONS THAT CAN BE PREVENTED
4. TO GET BETTER TREATMENT FOR CONDITIONS THAT I HAVE OR MAY DEVELOP
5. CONCERN FOR FAMILY MEMBERS
6. OTHER (SPECIFY)
7. DON’T KNOW
8. REFUSED

[Q: OTHERGP]

Prior to this survey, were you aware that national genome projects have been implemented in other countries such as Saudi Arabia and Kuwait?

1. YES
2. NO
3. DON’T KNOW
4. REFUSED

|

[Q: BEN_QATAR]

Aside from the personal benefits of genetic testing, collecting genetic information from all Qataris through the QGP, has added potential benefits for society as whole. Please rank them from first to last in order of importance. Which potential benefit would you say is the most important? [INTERVIEWER: REPEAT ITEMS AS NECESSARY]

1. Raising the international profile of Qatar
2. Contributing to a healthier population
3. Building an effective health care system though personalized treatment
4. DON’T KNOW
5. REFUSED
6. **Exit Demographics**

#### [Q: HHINCO1]

#### Thank you for your cooperation. We have only 2 more questions related to the total monthly income of your family. First, is it less than QR 50,000 or QR 50,000 or more?

1. LESS THAN QR 50,000
2. QR 50,000 OR MORE
3. DON’T KNOW
4. REFUSED

#### [Q: HHINCO1A]

***IF HHINCO1 =1, ASK***

Second, is it less than QR 30,000 or QR 30,000 or more?

- - 1. LESS THAN QR 30,000
    2. QR 30,000 OR MORE

1. DON’T KNOW
2. REFUSED

#### [Q: HHINCO1B]

***IF HHINCO1 =2, ASK***

Second, is it less than QR 70,000 or QR 70,000 or more?

- - 1. LESS THAN QR 70,000
    2. QR 70,000 OR MORE

1. DON’T KNOW
2. REFUSED

[Q: THANKS1]

Those are all the questions I have for you. Before I say good-bye, are there any other comments you'd like to make?

- 1. YES [OPEN-END]
  2. NO

Thank you very much for participating. We appreciate the time you have taken to complete this interview.

[READ IF NECESSARY:] If you have any questions on the purpose of this study, you can call my supervisor here at SESRI. We're at 4403- 3030 – just mention the telephone survey.

Again, thank you and goodbye.

[Q: THANKS2]

***IF RESPTYPE <> 1 OR CONFIRM <>1 , SAY***

Thank you very much for your time. Our questions would not apply to you but we appreciate your willingness to participate. Have a nice day.

**ITEMS FOR INTERVIEWER**

{Q: R_LANG}:

THE INTERVIEW WAS CONDUCTED IN…

1. ARABIC (QATARI DIALECT INTERVIEWER)
2. ARABIC (NON-QATARI DIALECT INTERVIEWER)
3. ENGLISH

{Q: IVCOM}

IV: PLEASE NOTE ANY PROBLEMS DURING THE INTERVIEW THAT WOULD AFFECT THE QUALITY OF THE DATA.

{Q: IVID}

IV: PLEASE PUT YOUR INTERVIEWER ID (NOT YOUR STATION NUMBER) HERE.
