# Supplementary material for: Willingness to participate in genome testing: a survey of public attitudes from Qatar
Source: J Hum Genet. 2020 Jul 28;65(12):1067–73. doi: 10.1038/s10038-020-0806-y (PMC7605429; doi:10.1038/s10038-020-0806-y)
Supplement: Supplementary file 2 — Supplementary Table [file 10038_2020_806_MOESM2_ESM.docx]

**Supplementary Table. Attitudes towards genomic testing by demographic characteristics of respondents**

|  | **Willing to participate in genomic testing (%)*** | ***P***** |
| --- | --- | --- |
| **Age** |  | 0.35 |
| **18-24** | 69.8 |  |
| **24-34** | 74.5 |  |
| **35-44** | 74.4 |  |
| **45+** | 67.7 |  |
| **Marital status** |  | 0.57 |
| **Married** | 72.5 |  |
| **Unmarried** | 68.6 |  |
| **Other** | 69.4 |  |
| **Blood related to spouse** |  | 0.31 |
| **Yes** | 74.7 |  |
| **No** | 70.5 |  |
| **Education** |  | 0.49 |
| **Less than secondary** | 68.2 |  |
| **Secondary or vocational** | 73.0 |  |
| **Undergraduate degree or above** | 69.7 |  |
| **Monthly household income** |  | 0.057 |
| **Less than ~$8,240** | 70.0 |  |
| **~$8,240 – $13,730 ***** | 81.5 |  |
| **~$13,730 – $19,230** | 70.1 |  |
| **More than ~$19,230** | 70.5 |  |

*Reported percentages were calculated using survey weights

** Based on a design-based F (a corrected weighted Pearson chi square statistic)

*** Willingness in this level of income was significantly different from other levels
